# Soft magnetic thin film deformation with a bistable electropermanent magnet

**DOI:** 10.1088/2631-8695/acf2e8

**Published:** 2023-09-13

**Authors:** Nolen I. Keeys, Dinesh K. Patel, Philip LeDuc, Carmel Majidi

**Affiliations:** 1 Department of Mechanical Engineering, Carnegie Mellon University, United States of America; 2 Human-Computer Interaction Institute, Carnegie Mellon University, United States of America

**Keywords:** magnet, soft, deformation, thin film, electropermanent, magnetorheological elastomer

## Abstract

Physically soft magnetic materials (PSMMs) represent an emerging class of materials that can change shape or rheology in response to an external magnetic field. However, until now, no studies have investigated using an electropermanent magnet (EPM) and magnetic repulsion to magnetically deform PSMMs. Such capabilities would enable the ability to deform PSMMs without the need for continuous electrical input and produce PSMM film deformation without an air gap, as would be required with magnetic attraction. To address this, we introduce a PSMM-EPM architecture in which the shape of a soft deformable thin film is controlled by switching between bistable on/off states of the EPM circuit. We characterized the deflection of a PSMM thin film when placed at controlled distances normal to the surface of the EPM and compared its response for cases when the EPM is in the ‘on’ and ‘off’ states. This work is the first to demonstrate a magnetically repelled soft deformable thin film that achieves two electronically-controlled modes of deformation through the on and off states of an EPM. This work has the potential to advance the development of new magneto-responsive soft materials and systems.

## 1. Introduction

Physically soft magnetic materials (PSMMs) that can undergo changes in shape, rheology, or local magnetic field with an applied stimulus have become an increasingly interesting area of research due to their implications in the growing fields of 4D printing, haptics, and soft robotics [1–4]. PSMMs are implemented using a variety of designs and form factors, including bulk magnets encapsulated in elastomers [5], micro- or nano-scale magnetic particles embedded in elastomers (e.g., magnetorheological elastomer (MRE)) [6–12], and micro- or nano-scale magnetic particles dispersed in water, oil, or an organic solvent (e.g., magnetorheological fluid (MRF)) [13–15]. When exposed to an externally applied magnetic field, often through the application of a permanent magnet (PM) or an electromagnet (EM), these materials experience mechanical or rheological changes where the magnitude of the material change is influenced by the intensity of the applied magnetic field [7]. This functional characteristic has enabled the development of new soft small-scale magnetic systems capable of sensing and actuation, with recent works demonstrating novel soft magnetic-based tactile sensors [12, 16], haptic devices [14, 17], and 4D printed structures [9, 11]. However, despite such progress in recent years, PMs and EMs still have several limitations. PMs typically require manual placement, and EMs depend on a continuous supply of high electrical current input that requires significant power and can result in Joule heating. Such constraints can be especially limiting for the development and application of PSMM systems and shape deformable surfaces.

Electropermanent magnets (EPMs) address many of the key challenges associated with utilizing PMs and EMs by eliminating the need for continuous electrical power to maintain a stable electromagnetically-controlled field. EPMs function as a dynamically tunable permanent magnet that can be effectively turned on and off with a pulse of current [18]. Recently, studies have utilized this bistable configuration of the EPM to magnetically compress microfluidic channels [19], control the flow of magnetorheological fluid [20], and achieve concave deformations of a soft magnetic membrane [21] at frequencies <1 Hz and with magnetic attractive forces. However, progress in coupling EPMs with PSMMs also depends on examining the role of magnetic repulsive forces in PSMM deformation. For example, magnetic repulsion can allow a magnetic material to deform outward and press against an external surface. It can also allow PSMMs to achieve new modes of shape change such as concave deformations as well as bidirectionality. For these reasons, magnetic repulsive forces generated by PM and EMs have been used to deform PSMMs [8, 16, 22–24]. This has enabled soft magnetic systems capable of complex shape changes and functionality such as pop-up 3D soft magnetic structures [9, 11], miniature swimming robots [23], and miniature soft robots capable of multimodal locomotion [10]. However, to our knowledge, no studies have investigated using EPMs to magnetically deform PSMMs with magnetic repulsion. Thus, the bistable configurations of an EPMs to magnetically repel a PSMM could lead to new classes of soft systems with a greater variety of stable deformation modes and less dependency on continuous electrical power input.

**Figure 1.**
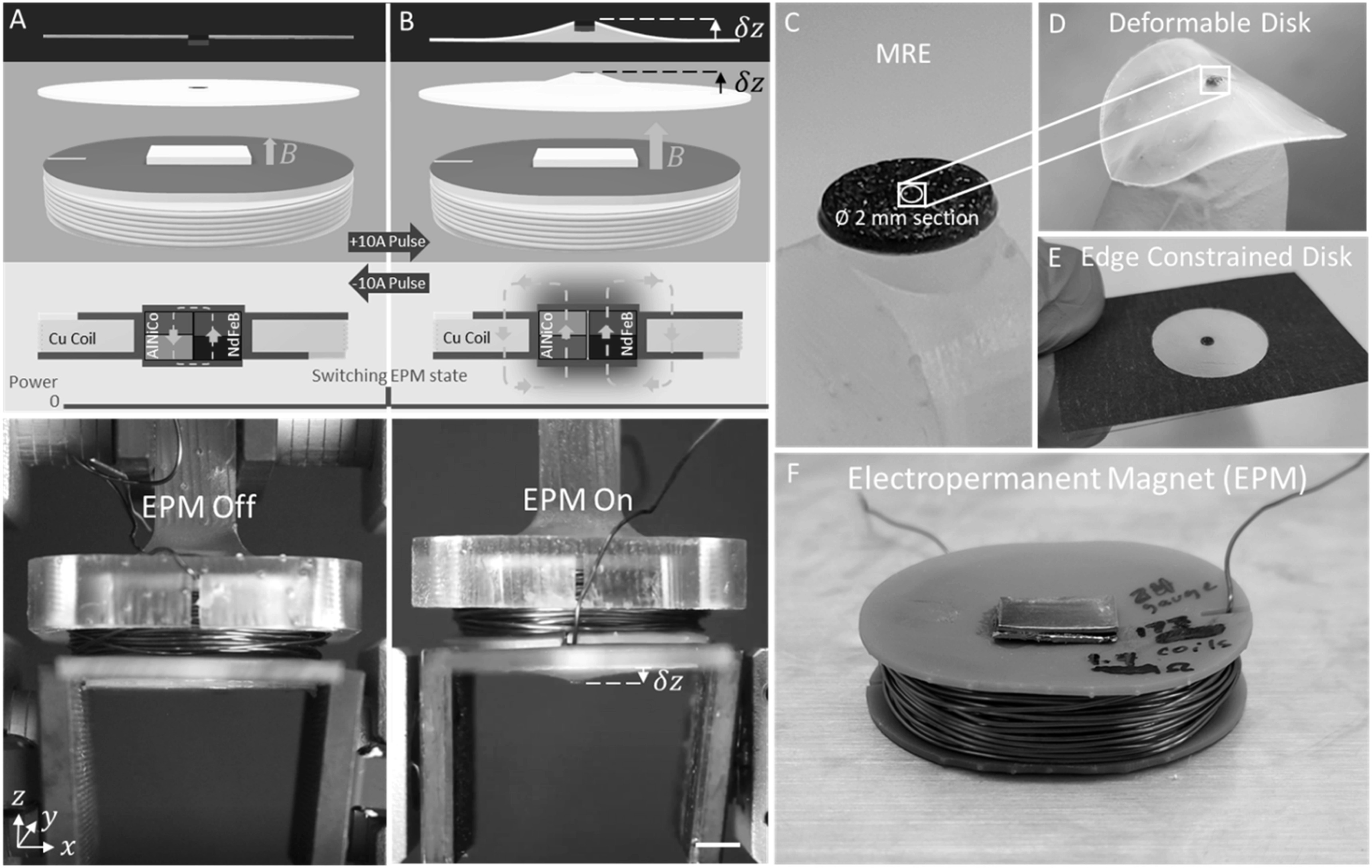
Deformation overview: (A) The thin film elastomer disk featuring an embedded small magnet is minimally repelled when placed normal to the surface of an electropermanent magnet (EPM) in the off state. In the EPM off state, the polarity of the AlNiCo and NdFeB magnets (which compose the EPM) are opposite each other. This causes the magnetic flux to preferentially flow inside of the EPM and results in a magnetic flux density in air surrounding the EPM that is insufficient to substantially repel the thin film. (B) When the EPM is in the on state, the small magnet embedded in the thin film is magnetically repelled producing an axially symmetric concave deformation of the disk. In the EPM on state, the polarity of the AlNiCo and NdFeB magnets are the same, causing the magnetic flux to preferentially flow outside of the EPM assembly, resulting in a magnetic field in the surround air that is greater than when the EPM is off. The switch between the two states is achieved by applying a ±10 A pulse of current to the coil surrounding the AlNiCo and NdFeB magnets. The scale bar is 5 mm. The embedded small magnet in the thin film is either a Ø1.59 mm rigid magnet or a (C) Ø2 mm section of magnetorheological elastomer (MRE). (D) The deformable disk is (E) centered and edge constrained on a glass slide featuring a Ø20 mm cutout then placed normal to the surface of the EPM.

Here, we present a magnetically repelled soft deformable thin film that achieves two electronically controlled configurations through zero-power bistable ‘on’ and ‘off’ states of an EPM. The thin film is composed of a Ø30 mm × 300 *μ*m thin elastic film with an embedded small permanent magnet. When the thin film is placed normal to the surface of the EPM in the off state such that the magnetic poles are opposite each other, the small magnet is minimally repelled (figure 1(A)). When the EPM is in the on state, the EPM magnetically repels the small magnet, deforming the thin film into an axially symmetrical concave shape that remains in a deformed state without the need for additional electrical power input until the EPM is switched off (figure 1(B)). The embedded small permanent magnet is either a rigid magnet or an MRE (figure 1(C)), that is encircled with a thin film of non-magnetic elastomer (figure 1(D)), edge constrained on a glass slide (figure 1(E)) and is deformed using an EPM with modified endcaps (figure 1(F)). We tracked the deflection of the small magnets embedded in the thin film as a function of distance from the EPM, in both the on and off states, and we observed a maximum average peak deflection magnitude of 0.706 mm when the EPM was in the ‘on’ state. In the ‘off’ state, the PSMM film had a maximum deflection of 0.358 mm. Furthermore, we applied a kinematic and a force model to examine the deflection profile of the deformable disk up to the point of peak deflection. We believe this work offers new insights, a potential new approach to deform PSMMs using magnetic repulsion, and could contribute to the development of energetically efficient shape morphing structures.

## 2. Methods

### 2.1. EPM construction

**Figure 2.**
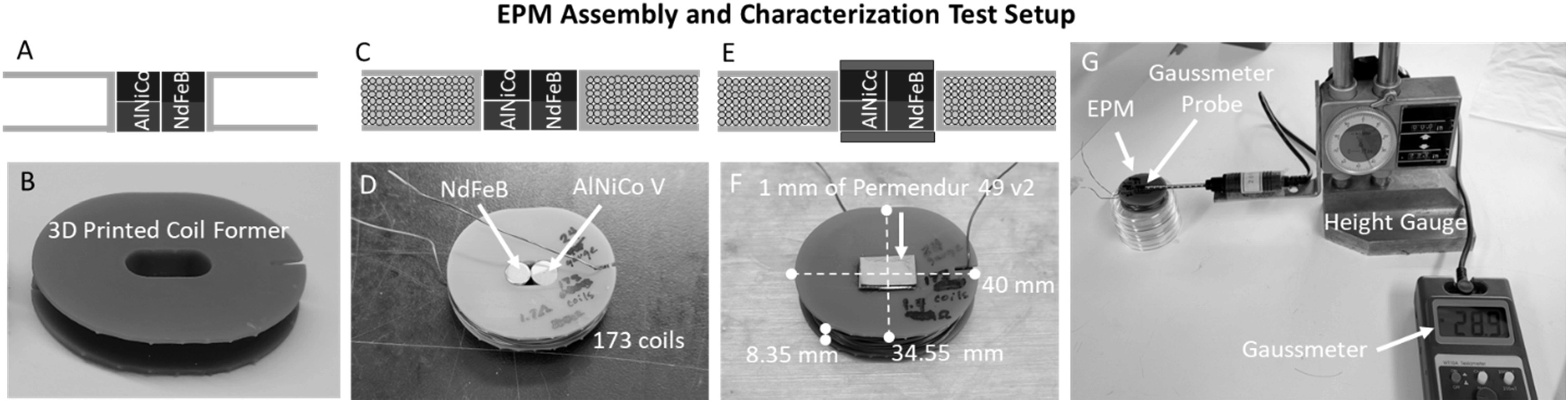
EPM assembly and characterization: (A)–(B) Two cylindrical axially magnetized AlNiCo and NdFeB magnets of similar size were placed inside of a 3D printed coil former. C-D) With the coil former, 173 coils of 24-gauge magnet wire were wrapped around the magnets and E-F) 1 mm of Permendur 49 v2 was used to magnetically connect the poles of the AlNiCo and NdFeB magnet. F) The dimensions of the final assembly were 40 mm × 34.55 mm × 8.35 mm. G) The magnetic flux density above the assembled EPM in both the EPM on state and the off state was measured using a gaussmeter and a height gauge.

The EPM was constructed according to the general fabrication guidelines presented by Knaian *et al* [18], but with changes to the endcaps to enable magnetic repulsion. Two cylindrical axially magnetized magnets were paired together in a 3D printed coil former, a low intrinsic coercivity magnet (H_ci_) (AlNiCo V, McMasterCarr Part#: 5852K13) and a high intrinsic coercivity magnet (NdFeB, K&J Magnetics Part#: D44) with similar residual inductances (figures 2(A)–(B)). The EPM assembly was then wrapped with 173 coils of 24-gauge magnet wire (K&J magnetics Part#: MW24-8) which was used to electronically generate a magnetic field pulse that controlled the polarity of the AlNiCo magnet (figures 2(C)–(D)). This enabled electronic control of the EPM states. 1 mm of high permeability material (*μ*r = 150, Goodfellow Permendur 49v2) was adhered to both magnetic poles of the magnet using cyanoacrylate adhesive to magnetically connect the poles of the magnets and complete the EPM assembly (figures 2(E)–(F)). Unlike with traditional EPMs, the endcaps were not extended beyond the magnets, which allowed us to maintain the location of the north and south poles along the axis of magnetization.

### 2.2. Electronic control of the EPM state

The state of the EPM was switched by applying a 1 s ±10 A pulse of current to the coils of magnet wire surrounding the EPM using a power supply (Eventek KPS3010D). The ±10 A of current supplied to coils generated a magnetic field inside of the coils that was greater than two times the intrinsic coercivity of AlNiCo. This produced a magnetizing field (~100 kA m^−1^) inside of the coil that was sufficient to switch the magnetic polarity of the AlNiCo magnet (H_ci_ ≈ 48 kA m^−1^), but not the NdFeB magnet (H_ci_ ≈ 950 kA m^−1^) [25]. Thus, a +10 A or a −10 A pulse of current was used to switch the EPM from ‘off’ to ‘on’ and from ‘on’ to ‘off,’ respectively, by switching the polarity of the AlNiCo magnet to have either the same or opposite polarity of the NdFeB magnet.

### 2.3. EPM characterization experimental setup

The magnetic flux density above the EPM was experimentally measured in the on state and the off state using a gaussmeter and a linear height gauge (ELEOPTION Tesla Meter Gauss Meter) (figure 2(G)). The gaussmeter probe was rigidly secured to the height gauge directly above the north pole of the EPM and the distance from the EPM was increased in increments of 0.05 in (1.27 mm), to 40 mm. At each step, the magnetic flux density was measured in Millitesla.

### 2.4. Assembly of the deformable disk

**Figure 3.**
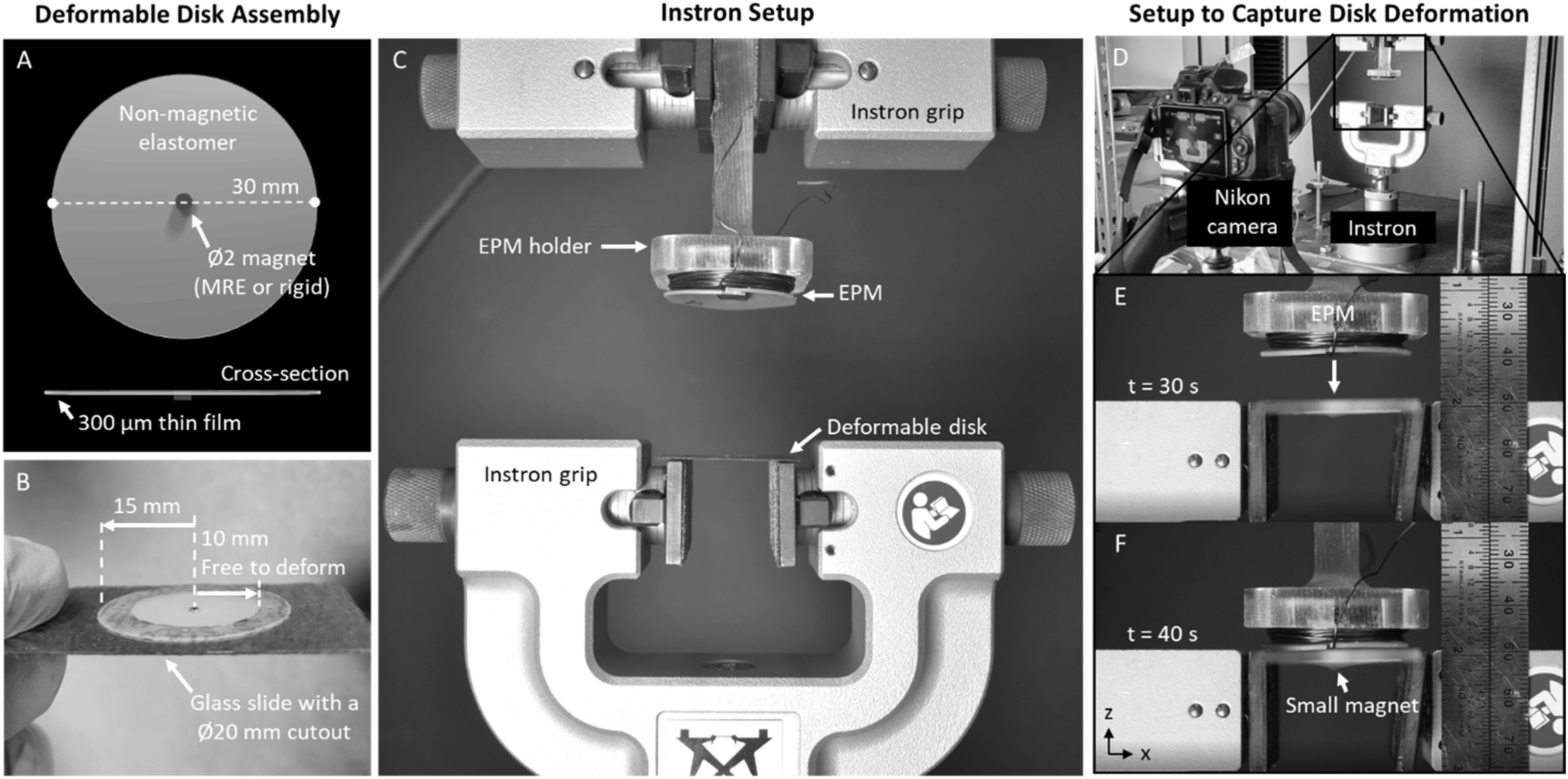
Deformable disk assembly and experimental setup to capture disk deformation: (A) The Ø30 mm × 300 *μ*m deformable disk features an embedded ~Ø2 mm magnet. (B) The deformable disk is edge constrained on a glass slide which has a Ø20 mm cutout so that a Ø20 mm section of the deformable disk is free to deform. (C) The EPM and the deformable disk were attached to the grips of an Instron tensile tester, and (D) a Nikon camera was used to record the deformation of the disk with respect to distance. The EPM was set at an initial distance of 40 mm from the deformable disk and the Instron was programmed to reduce this distance to 0 mm at a rate of 1 mm s^−1^. (E)–(F) Screen captured images show the movement of the EPM and the deflection of the deformable disk center as the distance is decreased, t = 30 s and t = 40 s.

The deformable disk is a Ø30 mm × 300 *μ*m axisymmetric elastomer thin film (Smooth-On Ecoflex 00-10) fabricated with an embedded axially magnetized permanent magnet at the center (figures 3(A) and S1). For testing, the deformable disk was placed on a glass slide such that the center Ø20 mm was free to deform and the edges were constrained (figure 3(B)). Three magnets were individually tested in the deformable disk that each vary in either size, composition, or magnetic volume to determine if the type of embedded magnet affects the response of the thin film. We tested one MRE (Ø2 mm, V_MRE_ = 3.14 mm^3^, figure S2) and two rigid magnets: the K&J Magnetics D101-N52 magnet (Ø1.59 mm, V_D101-N52_ = 1.59 mm^3^) and the K&J Magnetics D11-N52 magnet (Ø1.59 mm, V_D11-N52_ = 3.16 mm^3^). The permanently magnetized MRE was fabricated by curing and axially magnetizing a 1.2:1 vol. ratio (54.5% volume fraction) of Magnequench MQP 15-7 micro-sized ferromagnetic particles to Smooth-On Dragonskin 10NV (figures S1, S2).

### 2.5. Experimental setup to measure deformable disk deflection

The EPM was adhered to a non-magnetic holder using Scotch double-sided tape and secured upside down between the grips of an Instron tensile tester (figure 3(C)). Opposite the EPM, the deformable disk was placed and centered on a glass slide with a Ø20 mm cutout and the edges of the glass slide were adhered to the top of another set of grips such that Ø20 mm of the deformable disk was free to deform upward or downward. Both the EPM and the magnetic section of the deformable disk were arranged in this setup so that their north poles faced each other and the EPM was set at a distance of 40 mm above the deformable disk. This distance was sufficient for the magnetic fields of both magnets to not interact (decay to <0.1 mT). A camera captured images of the deformation of the disk (figure 3(D)) as the distance between the EPM and the deformable disk was decreased at a rate of 1 mm s^−1^ until the EPM was 0 mm above the glass slide (figure 3(E)–(F)). The displacement of the small magnets was measured using the video analysis software, Tracker, to quantify the deflection of the small magnets individually embedded in the deformable disks as a function of distance from the EPM. The programmed extension rate of −1 mm s^−1^ enabled tracking the deflection of the small magnets in EPM distance intervals of −0.167 mm.

## 3. Results

In this section, we assessed the bistable magnetic states of our EPM and investigated its interaction with our thin film to quantify the magnitude of deformation that is achieved with this approach. We (1) measured the magnetic flux density of the EPM at distances normal to its surface, and in both EPM states, which determined if the modification to the traditional EPM endcap enabled an EPM with bistable magnetic fields along its axis of magnetization, and (2) we quantified the deflection of the small magnets embedded in the deformable disk as a function of the EPM distance to investigate its interaction with the EPM in both of its states. Additionally, we (3) applied a kinematic and force model to model the displacement of the small magnet up to the point of peak deflection. These results are important because they provide insight into whether an EPM can be utilized for magnetic repulsion and can produce a deformation in our magneto-responsive thin film.

### 3.1. EPM characterization (Simulation)

**Figure 4.**
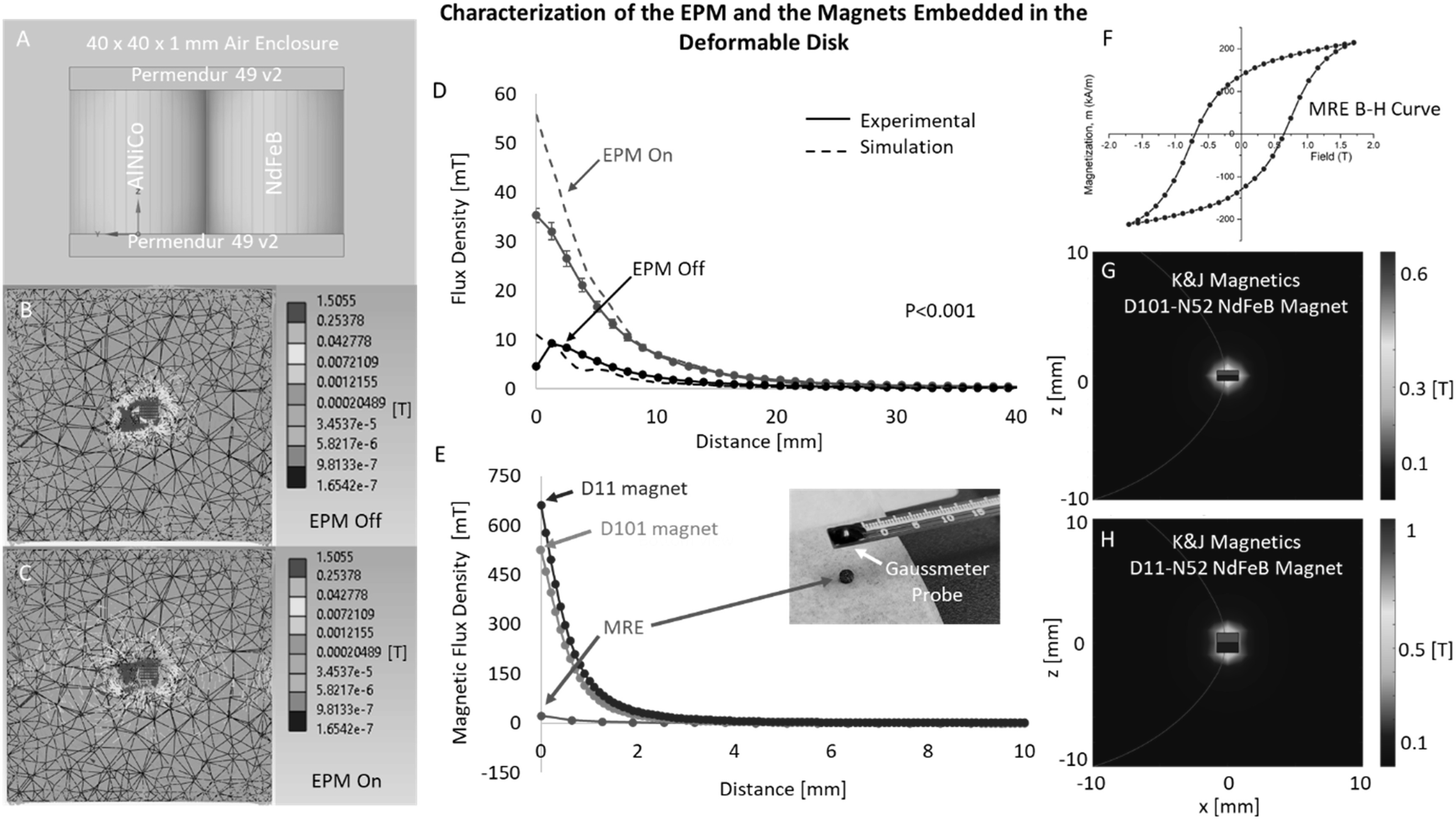
Characterization of the EPM and the magnets embedded in the deformable disk: (A) The EPM was modeled in, ANSYS, and a Magnetostatic Simulation Was Performed where the (B) EPM off state and the (C) EPM on state were both analyzed. The results show differences in the magnetic flux density along the EPM’s axis of magnetization between the two states. (D) The magnetic flux density along the EPM’s axis of magnetization was determined from this simulation and was plotted against the experimental measurements. Both the simulation results and the experimental measurements show the two distinct magnetic configurations of the EPM through the differences in the magnetic flux densities in the two states (P < 0.001). (E) The magnetic flux density along the axis of magnetization was experimentally measured for the MRE and determined using the Derby Olbert Model for Axially Magnetized Permanent Magnets for the rigid magnets. (F) The magnetization profile of the MRE in the form of a B-H curve was characterized by a vibrating sample magnetometer (VSM). 2D plots of the magnetic field for the (G) D101 and (H) D11 magnets were generated using the Derby Olbert Model to show magnet size, magnetic flux density, and axial symmetry.

The bistable magnetic states of the EPM were simulated using the multiphysics simulation software, ANSYS. In ANSYS, the EPM was represented in simulation based on its physical geometry and enclosed in a 40 × 40 × 1 mm air enclosure (isotropic *μ*_r_ = 1) (figure 4(A)). The magnetic properties of each component of the EPM were assumed to be: Permendur 49v2 (DC isotropic *μ*_r_ = 150) [26], AlNiCo (coercive force = 600 Oe, residual induction = 1.25 T) [18, 27], and NdFeB (coercive force = 11498 Oe, residual induction = 1.32 T) [28]. The results of the simulation generated a uniform vector plot of the magnetic field in the air enclosure surrounding the EPM in both the off state (figure 4(B)) and the on state (figure 4(C)). The magnetic flux density along the EPM’s axis of magnetization in both states were also determined from this simulation (figure 4(D)).

### 3.2. EPM characterization (Experimental)

Magnetic flux density measurements normal to the surface of the EPM quantifies the intensity of the magnetic field along the EPM’s axis of magnetization, which relates to the magnetic repulsive forces along this axis. Differences in the magnetic flux density measurements of the EPM when in the on state and the off state are therefore indicative of differences in the magnetic repulsive forces between the two states. With a gaussmeter, the magnetic flux density along the axis of magnetization of the EPM was experimentally measured in both the EPM on state and off state (see section 2.3). A peak average magnetic flux density of 35.24 mT in the EPM on state was measured directly on the surface (0 mm) and a peak average magnetic flux density of 8.58 mT in the EPM off state was measured at 1.67 mm above the EPM (figure 4(D)). We performed a Mann–Whitney rank-sum statistical test to determine that a statistically significant difference exists between the two EPM states (P < 0.001). Figure 4(D) is both the experimentally obtained magnetic flux density measurements and the results obtained from simulation. Both demonstrate the EPM’s bistable magnetic configurations.

### 3.3. Characterization of the magnets embedded in the deformable disk

Each of the magnets that were embedded in the deformable disks were characterized to quantify the magnetic flux density along their axis of magnetization (figure 4(E)) and their repulsive force in response to the EPM distance and state. For the MRE, the magnetic flux density along the axis of magnetization was measured experimentally (inset, figure 4(E)). Since the MRE is a composite, its magnetization was also characterized by a vibrating sample magnetometer (VSM) to determine its B-H curve (figure 4(H)). For the rigid magnets, the magnetic flux density along the axis of magnetization was determined using the Derby Olbert Model for Axially Magnetized Permanent Magnets (MATLAB: Magnetic Field Modeling package). These results are plotted along with the experimental values obtained for the MRE in figure 4(E). 2D magnetic field plots for the rigid magnets were also generated using the Derby Olbert Model to show the size of the D101 (figure 4(F)) and D11 (figure 4(G)) magnets, visualize their magnetic flux density, and visualize their axially symmetric magnetization. The repulsive force produced by each of the magnets embedded in the deformable disk, in response to the EPM distance and state, was quantified experimentally and is presented in figure S3.

### 3.4. Deformable disk deformation

**Figure 5.**
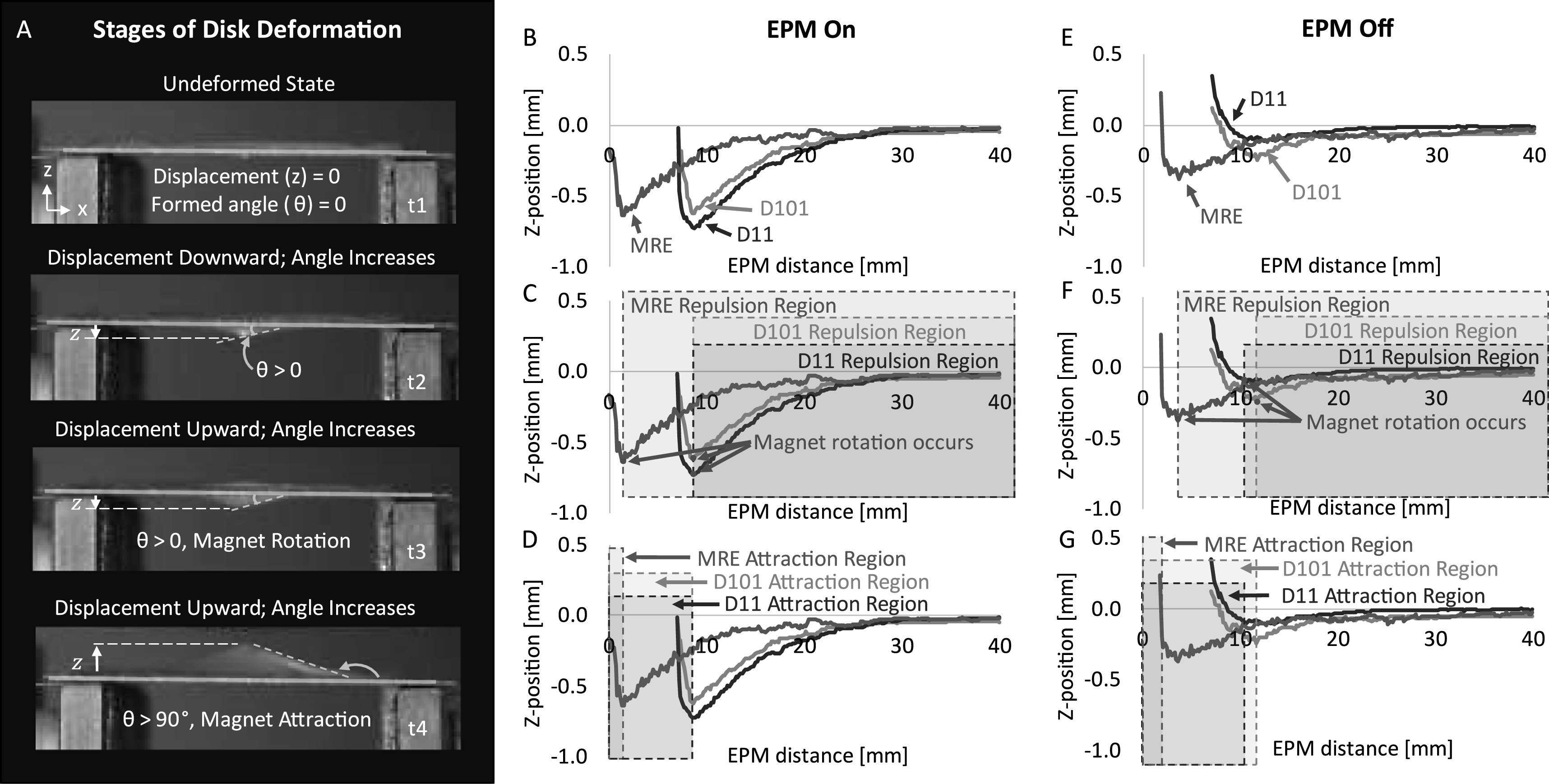
Disk deformation and embedded magnet deflection measurements: (A) As the EPM distance from the deformable disk was decreased, the embedded magnet displaced and deformed the thin film disk. The displacement of the embedded magnet was tracked until the EPM distance from disk was zero. (B) The average Z-position of the deformable disk center when the EPM was in the on-state was plotted with respect to distance. (C) The EPM distances where the embedded magnet was repelled in the on-state and (D) the EPM distances where the small magnet was attracted are indicated with a dashed bounding box. (E) The average Z-position of the deformable disk center when the EPM is in the off state was also plotted with respect to distance. Each magnet deflected less when the EPM was in the off state than when the EPM was in the on state. (F) The EPM distances where the small magnet was repelled in the off state and (G) the EPM distances where the small magnet was attracted are indicated with a dashed bounding box.

Measuring the displacement of the small magnet embedded in the deformable disk center at distances from the EPM quantified the deformation of the thin film with respect to the EPM distance. The deflection of the small magnet was determined by tracking the displacement of the embedded magnet (see section 2.6) as the disk deformed (figure 5(A)). This was repeated 10 times for each magnet, five times with the EPM on and five times with the EPM off, for a total of 30 tests. In the EPM on state, the deformable disk with the embedded MRE, D11, and D101 magnets produced an average peak deflection magnitude of 0.634 mm, 0.706 mm, and 0.575 mm, respectively (figure 5(B)). These peak deflections due to magnetic repulsion occurred when the EPM was on and at average distances of 1.5 mm, 8.67 mm, and 8.67 mm for the MRE, D11, and D101 magnets, respectively (figure 5(C)). At EPM distances less than the distance when peak deflection occurred, magnet rotation caused the magnetic pole on the opposite side of the magnet to attract to the EPM (figure 5(D)).

In the EPM off state, the deformable disk with the embedded MRE, D11, and D101 magnets produced an average peak deflection magnitude of 0.358 mm, 0.101 mm, and 0.191 mm, respectively (figure 5(E)). These peak deflections due to magnetic repulsion occurred when the EPM was at an average distance of 1.5 mm, 12.67 mm, and 11.67 mm for the MRE, D11, and D101 magnets, respectively (figure 5(F)). At EPM distances less than the distance when peak deflection occurred, magnet rotation caused the magnetic pole on the opposite side of the magnet to attract (figure 5(G)). These results are summarized in table 1.

**Table 1.** Summary of the average peak deflections for each magnet when the EPM is in the on/off state and the EPM distance at which this occurs. The difference between the peak deflections in the two states is also reported.

	EPM on		EPM off		
Small magnet	Average peak deflection magnitude [mm]	EPM distance at peak deflection [mm]	Average peak deflection magnitude [mm]	EPM distance at peak deflection [mm]	Difference in peak deflection magnitude [mm]
**D101** (V_D101-N52_ = 1.59 mm^3^)	0.575 ± 0.16	8.67	0.191 ± 0.08	11.67	0.384
**MRE** (V_MRE_ = 3.14 mm^3^)	0.634 ± 0.27	1.5	0.358 ± 0.21	3.67	0.276
**D11** (V_D11-N52_ = 3.16 mm^3^)	0.706 ± 0.17	8.67	0.101 ± 0.09	12.67	0.605

### 3.5. Modeling displacement with deflection angles

**Figure 6.**
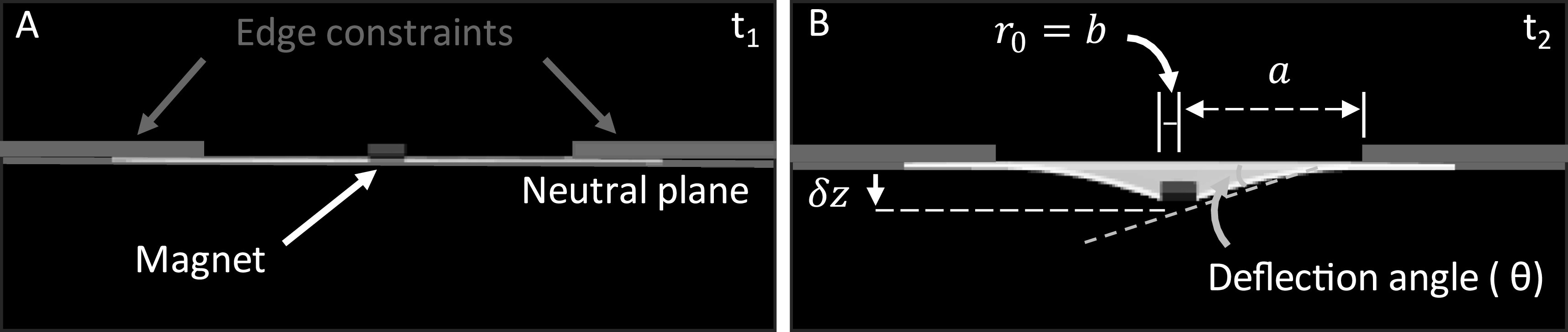
Schematic of the disk deformation and geometric inputs to the kinematic model: (A) At time t_1_, the deformable disk is centered, edge constrained on a glass slide, and undeformed. (B) At time t_2_, the embedded magnet is repelled by the EPM producing a displacement (z).

We implemented a kinematic model that described the relationship between the deformable disk deflection, deflection angle, and the EPM distance that will enable future design considerations with using magnetic repulsive thin films. The output of this kinematic model is displacement, and the deflection angle is the input. Accounting for the small strains, the equations from Young *et al* for an outer edge fixed, inner edge free circular plate of constant thickness [29] were used to model the displacement of the disk center, $z,$ as a function of the deflection angle ($\theta $) formed between the embedded magnet and the neutral plane of the deformable disk as the magnet deflected (figures 6(A)–(B)). The angle was measured using the same videos that captured the deflection of the small magnet and the software, Tracker. The equations are presented below where $a$ is the radius of the unconstrained disk, $b$ is the radius of the embedded magnet, $v$ is Poisson’s ratio = 0.5 (assuming incompressibility of the elastomer) [30], and ${C}_{1},$ ${C}_{4},$ ${L}_{1}$ and ${L}_{4}$ are geometric constants .

\begin{eqnarray*}z=\theta a\left(\frac{{C}_{1}{L}_{4}}{{C}_{4}}-{L}_{1}\right)\end{eqnarray*}

\begin{eqnarray*}{C}_{1}=\frac{1+v}{2}\frac{b}{a}\mathrm{ln}\frac{a}{b}+\frac{1-v}{4}\left(\frac{a}{b}-\frac{b}{a}\right)\end{eqnarray*}

\begin{eqnarray*}{C}_{4}=\frac{1}{2}\left[\left(1+v\right)\frac{b}{a}+\left(\left(1-v\right)\frac{a}{b}\right)\right]\end{eqnarray*}

\begin{eqnarray*}{L}_{1}=\frac{1+v}{2}\frac{{r}_{0}}{a}\mathrm{ln}\frac{{r}_{0}}{a}+\frac{1-v}{4}\left(\frac{a}{{r}_{0}}-\frac{{r}_{0}}{a}\right)\end{eqnarray*}

\begin{eqnarray*}{L}_{4}=\frac{1}{2}\left[\left(1+v\right)\frac{{r}_{0}}{a}+\left(1-v\right)\frac{a}{{r}_{0}}\right]\end{eqnarray*}

**Figure 7.**
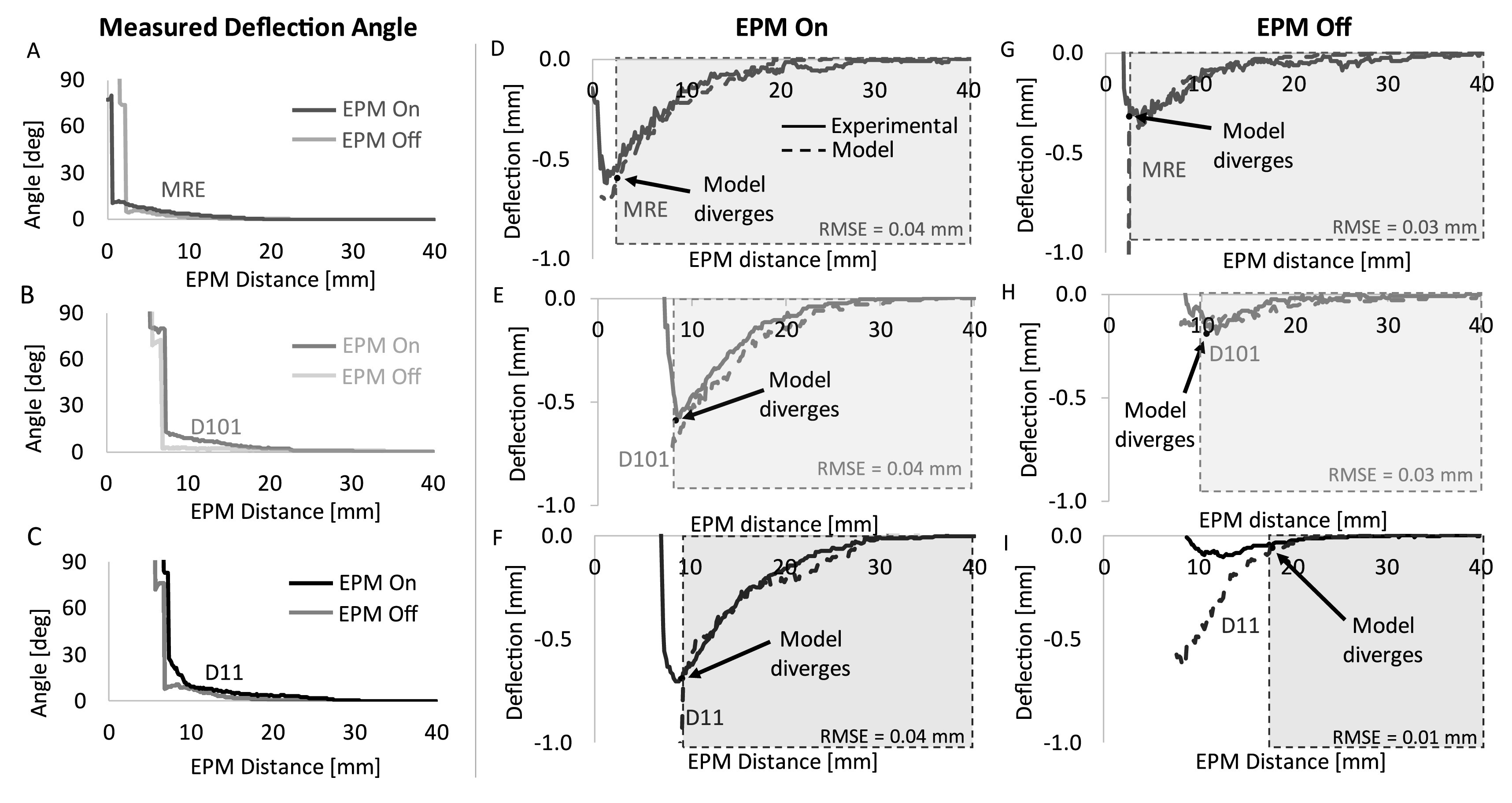
Kinematic model agreement with experimental data using deflection angle measurement: The deflection angle at various EPM distances was measured for the (A) MRE, (B) D101, and (C) D11 magnets in both the EPM states. These measured angles were used as the input to the kinematic model to assess the ability of the model to capture the kinematics of the disk deformation. The output of the kinematic was plotted against the directly measured deflection for each magnet when the EPM was in the (D)–(F) on state and the (G)–(I) off state. Up to the point of magnet rotation, the output of the model captured the deflection of the disk with a RMSE of <0.04 mm for each magnet and in both EPM states.

The deflection angles were measured for each embedded magnet and in both EPM states at each EPM distance (figures 7(A)–(C)). These experimentally obtained deflection angle measurements were then used in the kinematic model and the output of the model was compared with the experimentally measured displacement in both EPM states (figures 7(D)–(I)). These results assessed if the model captured the kinematics of the disk deformation. The agreement of the modeled deflection (using the deflection angle measurements) with the directly measured deflection was quantified using the statistical metric root mean squared error (RMSE). With the EPM in the on state, the deflection angles and the kinematic model captured the kinematics of the disk deflection with a RMSE of 0.04 mm all the magnets. In the EPM off state, the kinematics of the disk deflection were captured with a RMSE of 0.01 mm for the D11 magnet and 0.03 mm for the MRE and D101 magnets. The results of the experimental data and the model are discussed in section 4.3.

### 3.6. Modeling displacement with force

**Figure 8.**
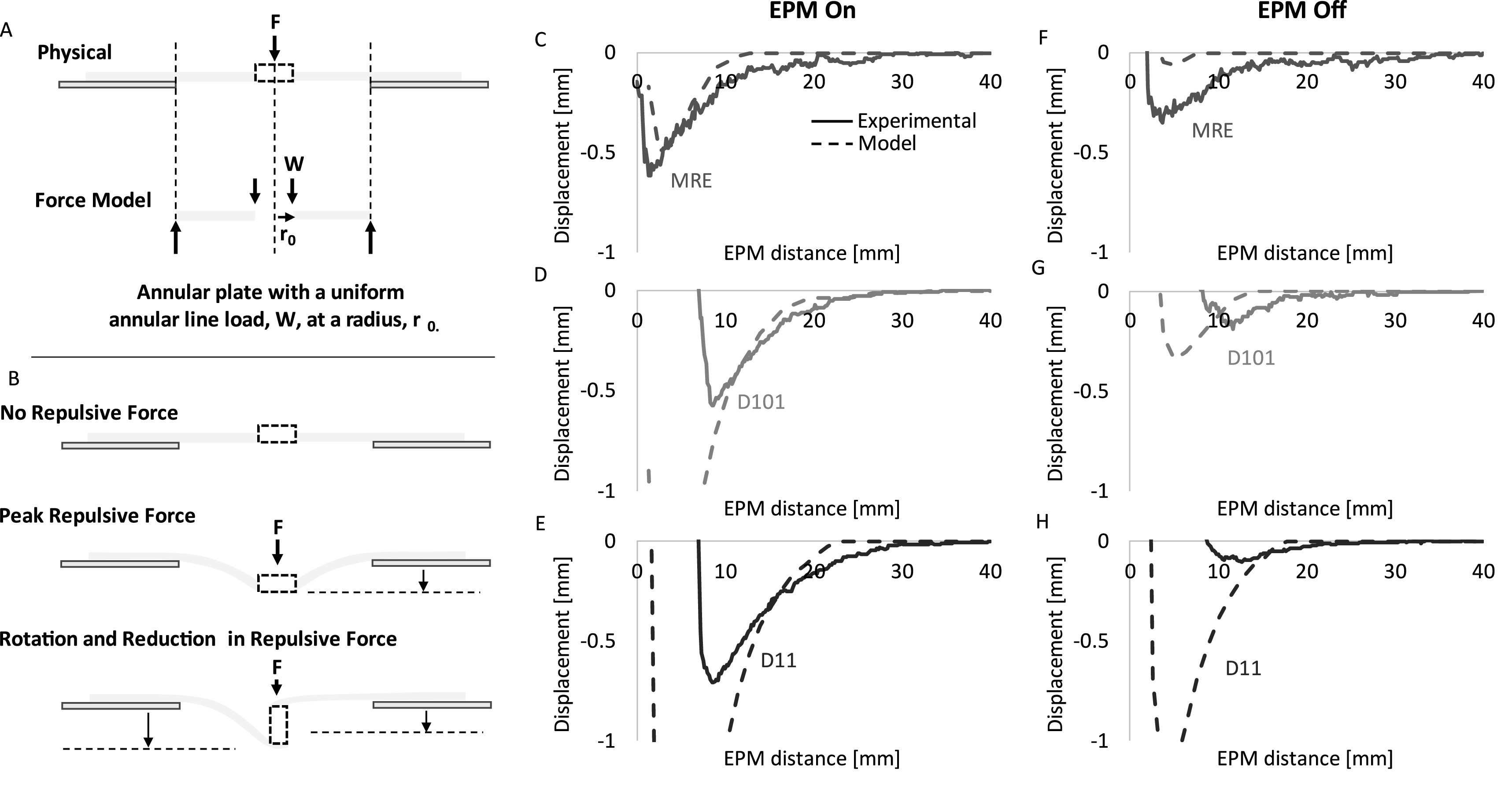
Force model agreement with experimental data using repulsive force measurements: The repulsive force produced by each of the magnets embedded in the deformable disk, when fully constrained and in response to the EPM distance and state (see figure S3), was used as the input to the force model to predict the deflection of the center of the deformable disk. (A) A schematic of the physical representation of the deformable disk is compared with a schematic of the force model applied which assumes a force is applied normal to the surface. However, (B) physically, when a force is applied to the deformable disk, the embedded magnet is repelled (negative displacement), the center of the disk displaces downward and then the embedded magnet rotates. In some cases, this rotation leads to a further increase in displacement if the radius of the magnet is larger than ½ its thickness (as is the case for the MRE and the D11 magnet). Thereafter, the magnet attracts to the surface of the EPM (positive displacement). The output of the force model is plotted against the directly measured deflection for each magnet when the EPM was in the (D)–(F) on state and the (G)–(I) off state.

The magnetic repulsive forces produced by the embedded magnets repelling from the EPM (see figure S3) were used as the input to a force model to predict displacement of the magnet in the deformable disk. We utilize a linear elastic force model for a simply supported annular plate of constant thickness with a uniform annular load line [29] (figure 8(A)) to describe the geometry and loading conditions of our deformable disk as it repels and deforms in response to a normal force (figure 8(B)). The equations for the force model are presented below where $a$ is the radius of the unconstrained disk, ${r}_{0}$ is the radius of the embedded magnet, $y$ is the displacement of the thin film, $w$ is the force, $D$ is the plate constant, $v$ is Poisson’s ratio = 0.5 (assuming incompressibility of the elastomer), $E$ is the Young’s modulus (50 kPA for Ecoflex 00-10) [31], $b$ is equal to ${r}_{0}$ (as the force is applied to the inner edge of the annular ring), and $t$ is the thickness of the thin film:

\begin{eqnarray*}y=\frac{-w{a}^{3}}{D}\left(\frac{{C}_{1}{L}_{9}}{{C}_{7}}-{L}_{3}\right)\end{eqnarray*}

\begin{eqnarray*}D=\frac{E{t}^{3}}{12(1-{v}^{2})}\,\end{eqnarray*}

\begin{eqnarray*}{C}_{1}=\frac{1+v}{2}\frac{b}{a}\mathrm{ln}\frac{a}{b}+\frac{1-v}{4}\left(\frac{a}{b}-\frac{b}{a}\right)\end{eqnarray*}

\begin{eqnarray*}{L}_{9}=\frac{{r}_{0}}{a}\left\{\frac{1+v}{2}\mathrm{ln}\frac{a}{{r}_{0}}+\frac{1-v}{4}\left[1-{\left(\frac{{r}_{0}}{a}\right)}^{2}\right]\right\}\end{eqnarray*}

\begin{eqnarray*}{C}_{7}=\frac{1}{2}\left(1-{v}^{2}\right)\left(\frac{a}{b}-\frac{b}{a}\right)\end{eqnarray*}

\begin{eqnarray*}{L}_{3}=\frac{{r}_{0}}{4a}\left\{\left[{\left(\frac{{r}_{0}}{a}\right)}^{2}+1\right]\mathrm{ln}\frac{a}{{r}_{0}}+{\left(\frac{{r}_{0}}{a}\right)}^{2}-1\right\}\end{eqnarray*}

Using the force data, the model predicts the displacement of the deformable disk center for each magnet and is compared with the experimentally obtained displacement data for each magnet when the EPM is on (figures 8(C)–(E)) and off (Figures (F)–(H)). In most cases, the force model predicts the displacement of the deformable disk within 0.2 mm, up to peak deflection. The results of the experimental data and the model are further discussed in section 4.3.

## 4. Discussion

This work is the first to investigate the use of an EPM, its bistable magnetic configurations, and magnetic repulsion to magnetically deform a soft, thin film with an embedded permanent magnet. The results of our experiments demonstrate that we (1) developed an EPM with bistable magnetic configurations along its axis of magnetization, (2) fabricated a deformable disk that can be magnetically repelled, (3) report the magnitude of deformation achieved with this approach, and (4) identified an approach to model the deformation of the disk for three different embedded small magnets in both EPM states. In this section, we statistically evaluate the bistable magnetic configurations of the EPM, compare the disk deflections produced with each embedded magnet in response to EPM distance and state, and discuss the kinematic model for modeling the deformation of the thin film.

### 4.1. Statistical test of the EPM’s bistable configurations

The Mann–Whitney rank-sum statistical test was applied to the experimentally obtained results that measured the magnetic flux density of the EPM, in both of its states, to test the null hypothesis that there was no statistically significant difference in the magnetic flux density along the EPM’s axis of magnetization (see figure 4(D)). We found our null hypothesis to be false, indicating that there was a change in the magnetic flux density above the EPM (P < 0.001). When the EPM was on, it produced an average peak magnetic flux density of 35.24 ± 1.38 mT, and in the off state, an average peak magnetic flux density of 8.85 ± 0.61 mT. These magnetic flux density measurements were predicted by the results of the ANSYS simulation where the largest absolute error was observed at the surface of the EPM and reduced at further distances.

### 4.2. EPM enables bistable magnetic repulsion of the embedded magnets

We investigated if magnetic repulsion and the bistable magnetic configurations of the EPM could be used to enable two magnetically controlled responses in the deformable disk. We found that each magnet embedded in the thin film could be repelled by the EPM in the on state and that this magnitude of deflection overall decreased when the EPM was in the off state (see figures 5(E)–(G)). Of the three magnets tested in the deformable disk, the D11 magnet had the greatest average peak deflection magnitude of 0.706 mm when the EPM was in the on state while the MRE and D101 rigid magnets had lesser peak deflection magnitudes given the same conditions, 0.634 mm and 0.575 mm, respectively (table 1). When the EPM was in the off state, the D11 magnet produced the least deflection of all three tested magnets, an average peak deflection magnitude of 0.101 mm. Alternatively, the MRE and D101 rigid magnets produced greater deflections than the D11 magnet when the EPM was off, despite the D11 being the strongest of the three magnets (see figure 4(E)). These results demonstrated that the embedded magnet with the greatest magnetic flux density had the greatest response to the bistable magnetic configurations of the EPM, producing the largest deformation when the EPM was on, and the smallest deformation was the EPM was off.

Considering that some applications might require an EPM at a fixed distance, we also investigated which embedded magnet would produce the greatest change in deformation due to an EPM state change at a fixed distance. We specifically considered the case of switching the EPM state from on to off at the point of peak deflection for each magnet, and we quantified the change in disk deformation as the percent change in magnet deflection (table 2). Of the three embedded magnets, the MRE produced the greatest percent change in deflection with an average peak deflection downward of 0.634 mm when the EPM was on, and an upward average peak deflection of 0.22 mm when the EPM was turned off. With the MRE, switching the EPM from on to off at the point of peak deflection (an EPM distance of 1.5 mm) would likely cause the MRE to attract to the EPM, cross the neutral plane of the deformable disk, and displace upward. However, this is not the case with the other embedded magnets. Changing the state of the EPM from on to off at the point of peak deflection for the D101 and D11 magnets would not on average cause the magnets to attract past the neutral plane of the deformable disk. Therefore, the D101 and D11 magnets are more ideal for achieving maximum deformation from magnetic repulsion when the EPM is on, and minimal magnetic repulsion when the EPM is off. These results also demonstrated the importance of considering the magnetic response of the deformable disk in both EPM states when at a fixed operating distance, to avoid any undesired responses. It is also important to note that increasing the EPM distance will likely decrease the percent change in deflection observed with changing the EPM state, but this leaves room for tuning the magnetic response of the thin film for a desired application.

**Table 2.** Summary of the average peak deflection for each magnet (when the EPM is on), the distance at which this peak deflection occurs, and the percent change in deflection if the EPM is switched off at this distance.

Small magnet	Average peak deflection magnitude when EPM is On [mm]	EPM distance at peak deflection [mm]	Deflection if EPM switched to off state [mm]	On–Off % change in deflection
**D101** (V_D101-N52_ = 1.59 mm^3^)	−0.575 ± 0.16	8.67	−0.2 ± 0.53	65.22%
**MRE** (V_MRE_ = 3.14 mm^3^)	−0.634 ± 0.27	1.5	0.22 ± 1.39	134.7%
**D11** (V_D11-N52_ = 3.16 mm^3^)	−0.706 ± 0.17	8.67	−0.005 ± 0.33	99.23%

### 4.3. Modeling the embedded magnet deflection

A model that captures the kinematics of the deformable disk deformation can be used to inform future design considerations (figure S4), thus we adopted a kinematic model and a force model that can be used to model the deflection of the thin film up to the point of magnet rotation for each magnet tested, and in both EPM states.

We found that the deflection angle formed between the embedded magnet and the neutral plane of the deformable disk could be used as an input to equation (1) to model the deflection of the magnet embedded in the thin film. With experimentally obtained deflection angles as the input to the model, we found that for each magnet and in both EPM states, the model captured the deflection of the disk with a RMSE of ≤0.04 mm, until the point of magnet rotation (see figures 7(D)–(I)). However, when the magnets rotated and displaced upward, the model diverged as the rotation of the magnet caused an increase in the angle, but no longer downward displacement (see figure 5(A)).

**Figure 9.**
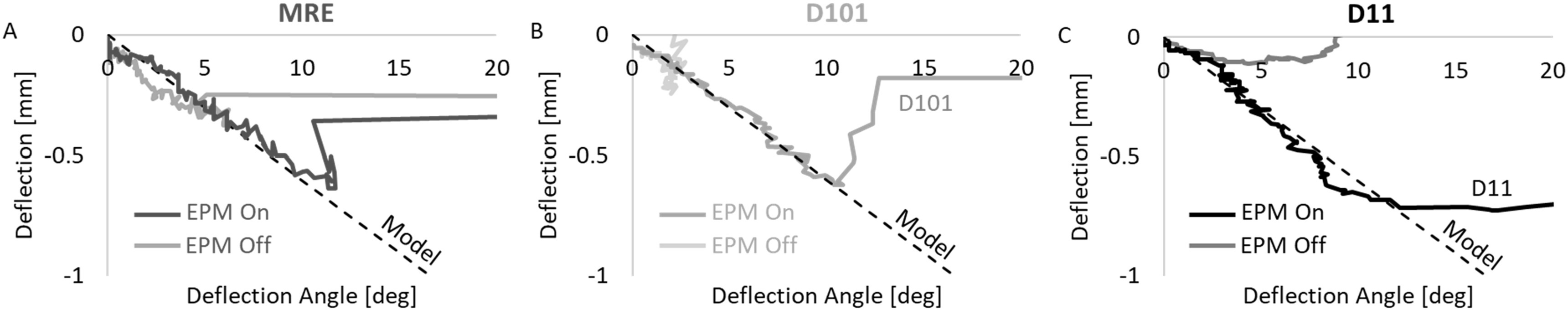
Experimental Data Compared with the Kinematic Model Output: Plots of the experimentally obtained deflection angles versus the deflection measurements plotted against the output of the kinematic model in both EPM states, and for each magnet, the (A) MRE, (B) D101, and (C) D11 magnets.

We then investigated the output of the model without the experimentally obtained deflection angles as input. We plotted the output of the kinematic model using angles from 0°–20° as the input and plotted the experimentally obtained deflection angles (*x*-axis) versus the experimentally obtained deflection measurements (*y*-axis) against it (figure 9). These plots show that the relationship between deflection angle and deflection can be described as a linear relationship that can be estimated using the kinematic model up to the point of magnet rotation, whereafter the model and the experimental results diverge (figures 7(D)–(I)). However, an approach is needed to predict when magnet rotation will occur. Until then, this model is most useful for estimating the disk deformation up to the point of magnet rotation.

We found that the magnetic repulsive forces produced by the interaction of the embedded magnets with the EPM can be used as the input to a force model to predict the deflection of the thin film for each magnet, and in both EPM states. We found that this model is promising for predicting the deflection of the thin film before the embedded magnet rotates, and unlike the kinematic model, it does not require one to assume a maximum formed angle, but in some cases it underestimates or overestimates the displacement of the embedded magnet.

The model predicts the displacement of each magnet embedded in the thin film and in each EPM state within 0.2 mm, up to the point when magnet rotation begins. For each of the characterized force interactions of the embedded magnets with the EPM, we observed largely magnetic repulsion but also magnetic attractive forces, which explains how the model predicts not only downward displacement but also upward (see figures S3 and 8). However, in some cases the model predicts the displacement of one magnet better than another.

In the case of the MRE and when the EPM is on, the model performs best, capturing the entire displacement curve including regions of repulsion, magnet rotation, and attraction within 0.1 mm. We determined that this is because the MRE does not always rotate when the EPM is on. Sometimes, the MRE only repelled, remaining close to or at its peak deflection when the EPM distance is zero. Therefore, the forces leading to displacement of the MRE mostly remained normal to the surface of the MRE, as it is an assumption of the model, and explains why the model agrees well with this case.

However, in all other cases, the displacement of the embedded magnet is predicted within 0.2 mm of the experimental data, but this difference increases when the magnet rotates and the assumption of the model that forces are normal to the surface of the magnet is no longer true. This is evident in the EPM on state for the D101 and D11 magnets just before peak deflection, where the magnet begins to rotate, and the model begins to overestimate the peak deflection. When the EPM is off, this is also evident with the MRE, D101, and D11 magnets as rotation occurs at EPM distances greater than when the EPM is on for each respective magnet leading to early divergence of the model. Notably, when the EPM is off, the model appears to underestimate the MRE and D101 displacement more than the D11 magnet. We determine that this is because the D11 magnet has a 1:1 aspect ratio so when it rotates in place about its central axis, its peak deflection does not significantly change. However, with the MRE and D101 magnets which have an aspect ratio of 2:1 and 1.58:1, respectively, rotation about a central axis may produce a peak deflection greater than just normal deflection (see figures 8(B), (F), and (G). We believe that this explains why the model underestimates the deflection of the MRE and D101 magnets when the EPM is off, more than with the D11 magnet.

Nevertheless, the proposed kinematic and force models can predict the deflection of each of the embedded magnet within a RMSE of ≤0.04 mm or <0.2 mm, respectively, but both do not fully capture the effect of the magnet rotation and attraction, except in the case of the force model when the EPM is on and with the embedded MRE. However, since this force model fully describes this one case where the assumptions of the model remain true, there is potential for this force model to fully describe the kinematics of this deformation if magnet rotation can be prevented or predicted.

### 4.4. Embedded magnet rotation leading to attraction

In the magnetic repulsion experiments, we consistently observed the embedded magnets rotating as the EPM distance continued to decrease after peak film deflection was achieved. This rotation would then lead to attraction of the small magnet and a reversal in the thin film deflection. As an example, when the EPM distance decreased at a rate of 1 mm s^−1^ past the point of peak deflection, the small magnets would slowly rotate, exposing the other side of the magnet to the magnetic field of the EPM and soon attract to the surface of the EPM. We suspect that this is result of magnetic torque described by the relationship $T={m}\times {B}$ where $T$ is the torque, $m$ is the magnetic moment of the small magnet, and B is the external magnetic flux density [32]. Magnetic moment, $m,$ is defined by the equation $m=\frac{1}{{\mu }_{0}}{B}_{r}V$ where ${\mu }_{0}\,$ is the magnetic permeability constant, ${B}_{r}$ is the residual inductance of the magnet, and $V$ is the volume of the magnet [32].

In figure S5, we present the results of torque calculations for the D101 and D11 magnets when the EPM is on and off which show that the D11 magnet would experience the greatest torque. Based on the calculations, the D11 magnet has the largest magnetic moment [28] and therefore, would experience the most torque when compared to the other magnets. Furthermore, these plots show that the torque increases substantially for each of the curves when the EPM distance is < 10 mm, the same region where peak deflection often occurred for the D11 magnet, suggesting that torque in this region overcomes the mechanical resistance of the thin film.

## 5. Conclusion

In this work we presented the first example of a magnetically repelled soft deformable thin film that achieves two magnetically-controlled responses through the zero-power bistable ‘on’ and ‘off’ states of an EPM. Through our modification to the traditional endcap geometry of an EPM, we enabled an EPM capable of magnetic repulsion. We then measured the magnetic flux density above the EPM in both of its states (which showed two bistable states like our simulation) and applied the Mann–Whitney rank sum statistical test which determined that there was a statistically significant difference between the two EPM states. We then utilized this EPM, and three small permanent magnets individually embedded in an axially symmetric thin film to produce two zero-power magnetically-controlled responses. We quantified these magnetic responses and determined a maximum deflection magnitude of 0.706 mm and a maximum decrease in deflection of the embedded magnet by 134.7% when the EPM is in the off state, when compared to the on state. With the kinematic and force models we proposed an approach to model the deflection of the thin film up to the point of magnet rotation for each magnet and both EPM states.

We believe this work has potential use in applications that require elastic materials to reversibly deform into two zero-power configurations or exhibit two zero-power magnetically-controlled responses. Specifically, since the EPM state produces two modes of deformation in the thin film, this approach may be useful for the development of soft switches, actuators, or haptic devices. Additionally, as force on the thin film varies with the proximity of the EPM and its state, this approach may be useful for industrial applications that require active dampening of thin film/membrane like structures. Future work may include working towards better understanding and predicting the critical distance where the magnets begin to rotate and then attract, exploring the intermediate magnetic configurations of the EPM that result from partially magnetizing the AlNiCo magnet and this effect on thin film deformation, and decreasing the bulk and size of the EPM. Additionally, decreasing the bulk and size of the EPM may prove this approach useful for the actuation of micro- or nano-scale devices.

## Data Availability

The data cannot be made publicly available upon publication because no suitable repository exists for hosting data in this field of study. The data that support the findings of this study are available upon reasonable request from the authors.

## Conflict of interest statement

The authors declare no conflict of interest.

## Ethics statement

This article does not contain any studies involving human participants performed by any of the authors.
